# Hospitalization Outcomes and Comorbidities of Bulimia Nervosa: A Nationwide Inpatient Study

**DOI:** 10.7759/cureus.2583

**Published:** 2018-05-05

**Authors:** Rikinkumar S Patel, Baris Olten, Priya Patel, Kaushal Shah, Zeeshan Mansuri

**Affiliations:** 1 Department of Global Public Health, Arcadia University, Philadelphia, USA; 2 Yale Child Research Center, Yale University School of Medicine; 3 Department of Psychiatry, Windsor University School of Medicine, Philadelphia, USA; 4 Psychiatry and Behavioral Health, Chicago Lakeshore Hospital; 5 Psychiatry, Texas Tech University Health Sciences Center at Odessa/permian Basin

**Keywords:** bulimia, eating disorders, comorbidities, national inpatient sample, inpatient psychiatry, demographics

## Abstract

Objective

To evaluate inpatient outcomes and the prevalence of psychiatric and medical comorbidities in bulimia nervosa.

Methods

We used the Nationwide Inpatient Sample (NIS) from the Healthcare Cost and Utilization Project (HCUP). We identified bulimia nervosa as the primary diagnosis and medical and psychiatric comorbidities using ICD­9-CM codes. The differences in comorbidities were quantified using the Chi-square (χ2) test, and a multinomial logistic regression model was used to quantify associations among comorbidities (odds ratio (OR)).

Results

The sample consisted of 3,319 inpatient admissions with bulimia nervosa between 2010–2014. Overall, 88% patients were younger than 40 years of age (p < 0.001). Bulimia nervosa was seen in a higher proportion of females (92.5%). The mean inpatient stay was 9.15 days and had a variable trend, whereas inpatient charges have been increasing (p < 0.001), averaging $34,398 (USD). The odds of having a longer hospitalization > 7 days (median) was seen in patients with comorbid fluid/electrolyte disorders (OR = 1.816; p < 0.001) and comorbid depression (OR = 1.745; p < 0.001). The most prevalent psychiatric comorbidities were psychosis (52.4%), followed by depression (23.5%). Females had three times higher odds of comorbid diabetes (OR = 3.374; p < 0.001), hypertension (OR = 2.548; p-value < 0.001), comorbid depression (OR = 1.670; p = 0.002), and drug abuse (OR = 2.008; p < 0.001).

Conclusion

Our study established psycho-socio-demographic characteristics, hospitalization outcomes, and comorbidities of bulimia nervosa patients. We believe that medical and psychiatric comorbidities of bulimia nervosa should be carefully investigated by clinicians as they can further complicate the management of bulimia nervosa and result in adverse inpatient outcomes.

## Introduction

Bulimia nervosa is a potentially life-threatening eating disorder characterized by recurrent episodes of binge eating and inappropriate compensatory purging behaviors, such as self-induced vomiting, laxative abuse, enemas, and excessive exercise [1]. World Health Organization World Mental Health Surveys estimated that the lifetime prevalence of bulimia nervosa is 0.8% and the median age of onset is in the late teens to the early twenties [2]. In the United States, the lifetime prevalence of bulimia nervosa in men and women was estimated to be 0.5% and 1.5%, respectively [3]. Bulimia nervosa has a significant impact on psychosocial functioning. In the United States, impaired psychosocial functioning was found among 78% of the patients; 44% reporting severe impairment.

Patients with bulimia nervosa usually have a lifetime history of many psychiatric disorders that were shown to be associated with a poorer prognosis [4]. The most common psychiatric co-morbidities of bulimia nervosa are unipolar major depression (50%), specific phobia (50%), post-traumatic stress disorder (PTSD) (45%), attention-deficit/hyperactivity disorder (ADHD) (35%) and alcohol use disorder (34%) [3]. Identifying and addressing the psychiatric co-morbidities of bulimia nervosa are crucial steps in the management of this disease as these co-morbidities can further decrease the psychosocial functioning. Suicide attempts are common with bulimia nervosa and were estimated to occur in 17% of the patients [5]. Successful treatment of bulimia nervosa can often resolve anxiety and depressive disorders [4]. Likewise, identifying and managing comorbid psychiatric disorders improve the treatment adherence and the outcome of treatment of bulimia nervosa.

Medical complications in patients with bulimia nervosa are common and can affect many organ systems, such as gastrointestinal, renal, cardiac, and endocrine [6]. Medical complications can contribute to a reduced quality of life and increased rates of mortality associated with bulimia nervosa. Compared to the general population, all-cause mortality is two to eight times greater in patients with bulimia nervosa [7]. Inpatient treatment of bulimia nervosa can be indicated in patients that have severe symptoms, psychiatric co-morbidities, or patients who develop serious medical complications [8].

In this inpatient sample-based retrospective study, we demonstrated the estimation of inpatient admission outcomes, as well as the rates of comorbid psychiatric and medical disorders in admitted bulimia nervosa patients. To the best of our knowledge, this is the first cohort retrospective study that investigates the inpatient psycho-socio-demographic characteristics and admission outcomes of bulimia nervosa patients.

## Materials and methods

Data source

In this study, a retrospective analysis was performed using the Healthcare Cost and Utilization Project's (HCUP) Nationwide Inpatient Sample (NIS) data [9]. The Agency for Healthcare Research and Quality (AHRQ) sponsors the HCUP databases that categorize trends in hospital utilization and cost across the United States. The HCUP-NIS database is the largest inpatient database as it includes 4,411 non-federal community hospitals from 45 states in the United States. We weighted the estimated samples to reduce the margin of error and to reproduce the results equivalent to all 50 states in the United States. In the NIS data, the privacy of individual patients, physicians, and hospitals, the state, and hospital identifiers are protected and de-identified. Sample non-clinical-related information is patient’s demographic data that includes age, gender, race and primary payer status, hospital characteristics, and total charges. Sample clinical-related information includes principal and other diagnosis, comorbidities, disposition status and the length of inpatient stay [9]. As the NIS database does not contain patient identification, this study did not require Institution Review Board permission.

Variables of interest

The participants in this study were identified by the International Classification of Disease, Ninth Revision Clinical Modification (ICD-9-CM) codes with a principal diagnosis of bulimia nervosa at the time of admission. Bulimia nervosa was identified using the ICD-9-CM diagnosis code 307.51. To measure the differences in hospitalization outcomes in bulimia nervosa patients, the outcome variables of interest included the severity of morbidity that measures the loss of body functions, the risk of mortality that measures the likelihood of dying, and discharge status of the patient. The 3M™ All Patient Refined Diagnosis-related Groups (APR-DRGs) were assigned using software developed by the 3M Health Information Systems (3M Health Information Systems, Murray, UT). This severity measure includes the APR-DRG, the severity of morbidity, and the risk of mortality within each base APR-DRG [9]. We calculated the length of the inpatient stay as the number of nights the patient remained in the hospital for a particular discharge. The length of inpatient stay in this analysis was all-cause. The total inpatient charges did not include professional fees and non-covered charges. The principal diagnosis had been identified at the time of admission, whereas we recorded comorbidities throughout the entire inpatient stay. By a common definition, comorbidities were considered coexisting conditions with bulimia nervosa, the principal disorder under this study. The Agency for Healthcare Research and Quality (AHRQ) comorbidity software was used to generate binary variables that identified various psychiatric and medical comorbidities in the discharge records using ICD-9-CM codes [10]. The ICD-9-CM codes used to identify comorbidities associated with bulimia nervosa are mentioned in Table 1.

**Table 1:** International Classification of Disease, Ninth Clinical Modification (ICD-9-CM) Codes Used to Identify Comorbidities of Bulimia Nervosa

Comorbidity	ICD-9-CM Diagnosis Code
Congestive heart failure	398.91, 402.01, 402.11, 402.91, 404.01, 404.03, 404.11, 404.13, 404.91, 404.93, 428.0-428.9
Hypertension	401.1, 401.9, 642.00-642.04, 401.0, 402.00- 405.99, 437.2, 642.10-642.24, 642.70-642.94
Diabetes without chronic complications	249.00-249.31,250.00-250.33,648.00-648.04
Diabetes with chronic complications	249.40-249.91,250.40-250.93,775.1
Hypothyroidism	243-244.2, 244.8, 244.9
Renal failure	403.01, 403.11, 403.91, 404.02, 404.03, 404.12, 404.13, 404.92, 404.93, 585.3, 585.4, 585.5, 585.6, 585.9, 586, V42.0, V45.1, V45.11, V45.12, V56.0-V56.32, V56.8
Obesity	278.0, 278.00, 278.01, 278.03, 649.10 - 649.14, 793.91, V85.30 - V85.39, V85.41 - V85.45, V85.54
Weight Loss	260 - 263.9, 783.21, 783.22
Fluid and electrolyte disorders	276.0 - 276.9
Deficiency anemias	280.1 - 281.9, 285.21 - 285.29, 285.9
Alcohol abuse	291.0 - 291.3, 291.5, 291.8, 291.81, 291.82, 291.89, 291.9, 303.00 - 303.93, 305.00 - 305.03
Drug abuse	292.0, 292.82 - 292.89, 292.9, 304.00 - 304.93, 305.20 - 305.93, 648.30 - 648.34
Psychosis	295.00 - 298.9, 299.10, 299.11
Depression	300.4, 301.12, 309.0, 309.1, 311

Approaches

We used the Statistical Package for the Social Science (SPSS), version 23.0 (IBM SPSS Statistics, Armonk, NY) to conduct a retrospective analysis over the HCUP-NIS database from 2010–2014 [11]. Descriptive statistics were used to summarize the results. The mean and standard deviations were used to explain the continuous variables. Pearson’s chi-square test and independent sample T-test were used for categorical data and continuous data, respectively. On the other hand, the categorical variables were presented in percentage (%) values. We used a multinomial logistic regression model to quantify associations among comorbidities and gender (odds ratio (OR); 95% confidence interval (CI)), and the inpatient length of stay (OR; 95% CI). We applied the discharge weight, which was given in the NIS database to attain national representation of the inpatient data. A p-value < 0.05 was used as a reference to determine the statistical significance test result.

## Results

Demographic characteristics of bulimia nervosa

**Figure 1:**
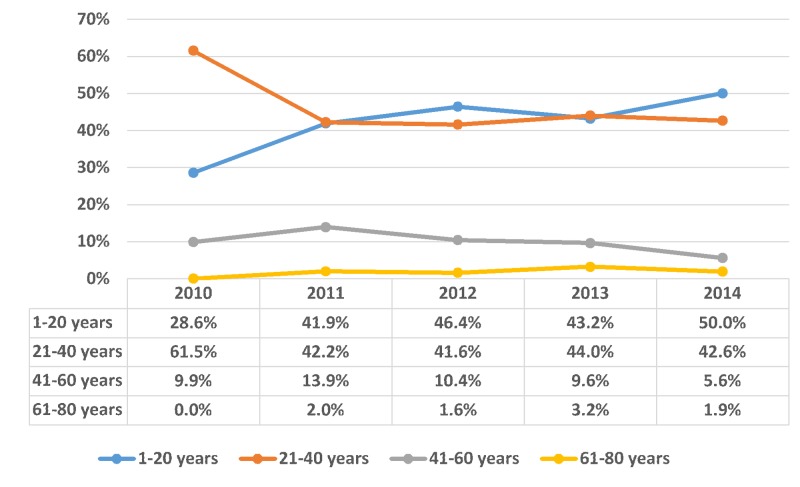
Trend of bulimia nervosa distributed according to age group X-axis: years; Y-axis: proportion in percentage (%). Significant p-values ≤ 0.05 at 95% confidence interval

**Figure 2:**
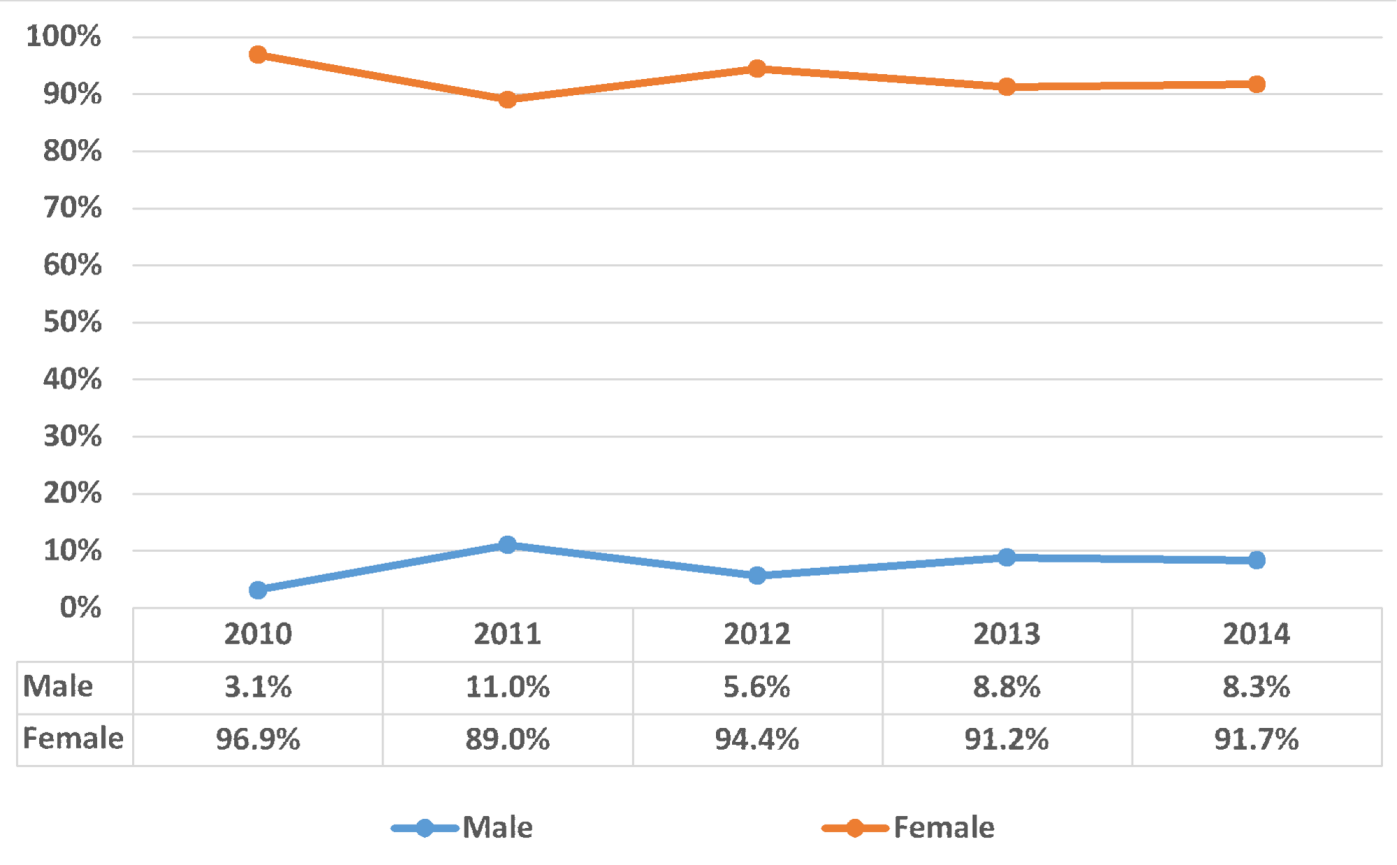
Trend of bulimia nervosa distributed according to gender X-axis: years and Y-axis: proportion in percentage (%); Significant p-values ≤ 0.05 at 95% confidence interval

The sample consisted of 3,319 inpatient admissions with bulimia nervosa listed as the primary diagnosis between 2010–2014. Overall, 46.5% patients were aged 21 - 40 years and 41.5% were under 20 years; thus, approximately 88% of bulimia nervosa patients were younger than 40 years of age (p-value < 0.001). On the contrary, bulimia nervosa was seen in 11.9% patients above 40 years’ age. Bulimia nervosa was seen in a higher proportion of females compared to the males (92.5% vs. 7.5%, respectively; p-value < 0.001). The trend of bulimia nervosa from 2010-2014 was distributed according to patient’s age group, as shown in Figure 1 with gender as shown in Figure 2.

**Figure 3:**
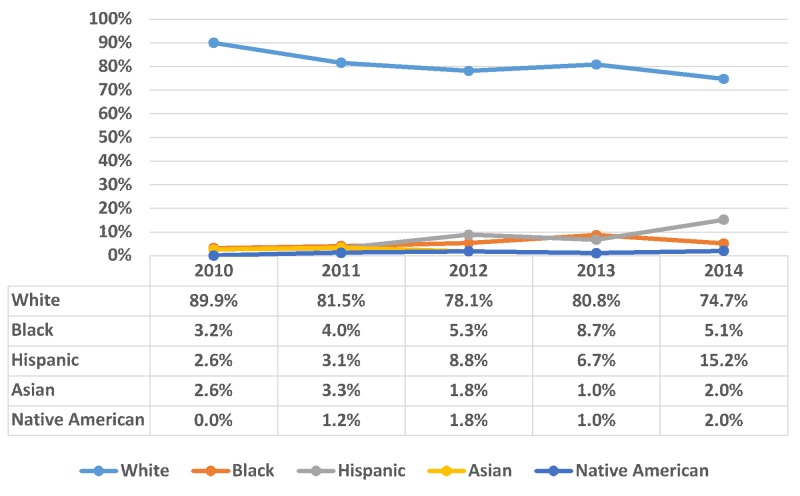
Trend of bulimia nervosa distributed according to race within bulimia nervosa patients X-axis: years; Y-axis: proportion in percentage (%). Significant p-values ≤ 0.05 at 95% confidence interval

A total of 2,457 bulimia nervosa patients were White, which comprises 81.4% of the total, followed by Hispanic (6.7%), Black (5%), Asian (2.3%), Native American (1.2%), and others (3.5%). The majority of the inpatient admissions for bulimia nervosa were covered by private insurance (55.7%) and Medicaid (23.8%). The trend of bulimia nervosa from 2010-2014 was distributed according to the patients’ race as shown in Figure 3.

Inpatient outcomes in bulimia nervosa

**Figure 4:**
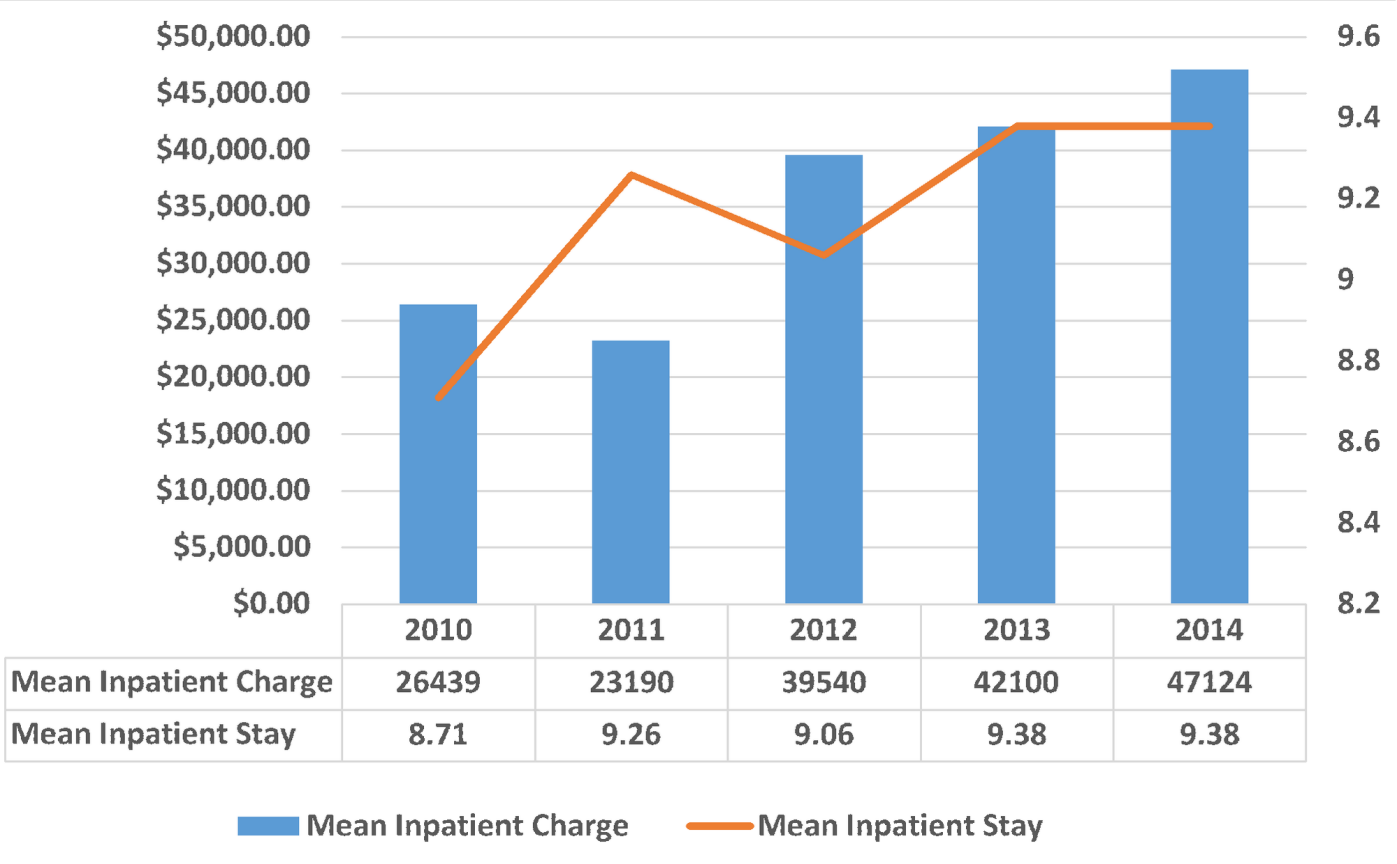
Trend of bulimia nervosa distributed according to the mean inpatient stay and mean inpatient charges X-axis: years; Left Y-axis: mean inpatient charge (in USD); Right Y-axis: mean inpatient stay (in days). Significant p-values ≤ 0.05 at 95% confidence interval. USD: United States dollars

A total of 2,428 patients (73.3%) were hospitalized based on non-elective admission and 253 patients (7.7%) with a principal diagnosis of bulimia nervosa were transferred in from an acute care facility. The mean length of stay in the hospital was 9.15 days (standard deviation (SD) = 7.80; p-value < 0.001), and the mean inpatient total charge was $34,398 USD (SD = 37692.19; p-value < 0.001). The trend of bulimia nervosa was distributed according to the mean inpatient stay and mean inpatient charges, as shown in Figure 4. During the period 2010–2014, there were a total of 30,367 inpatient days and $113 million USD inpatient costs due to bulimia nervosa in the United States.

**Figure 5:**
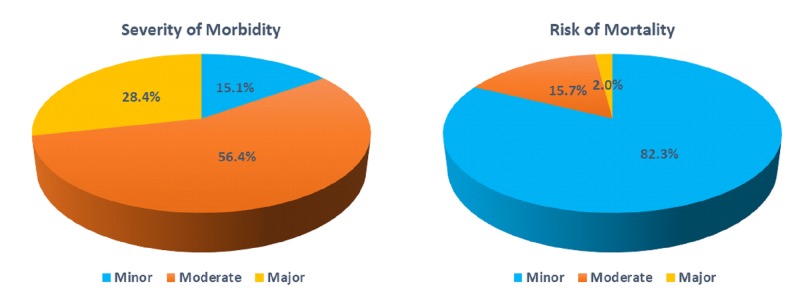
Distribution of bulimia nervosa according to the severity of morbidity and risk of mortality Significant p-values ≤ 0.05 at 95% confidence interval

About, 71.5% (number (N) = 2,375) of the bulimia nervosa patients had minor to moderate severity of morbidity and 98% (N = 3,254) patients were sub-classified as minor to moderate risk of mortality. There was no bulimia nervosa-related inpatient deaths between 2010–2014 in the United States hospitals. Figure 5 shows the severity of morbidity and risk of mortality in the bulimia nervosa patients.

**Figure 6:**
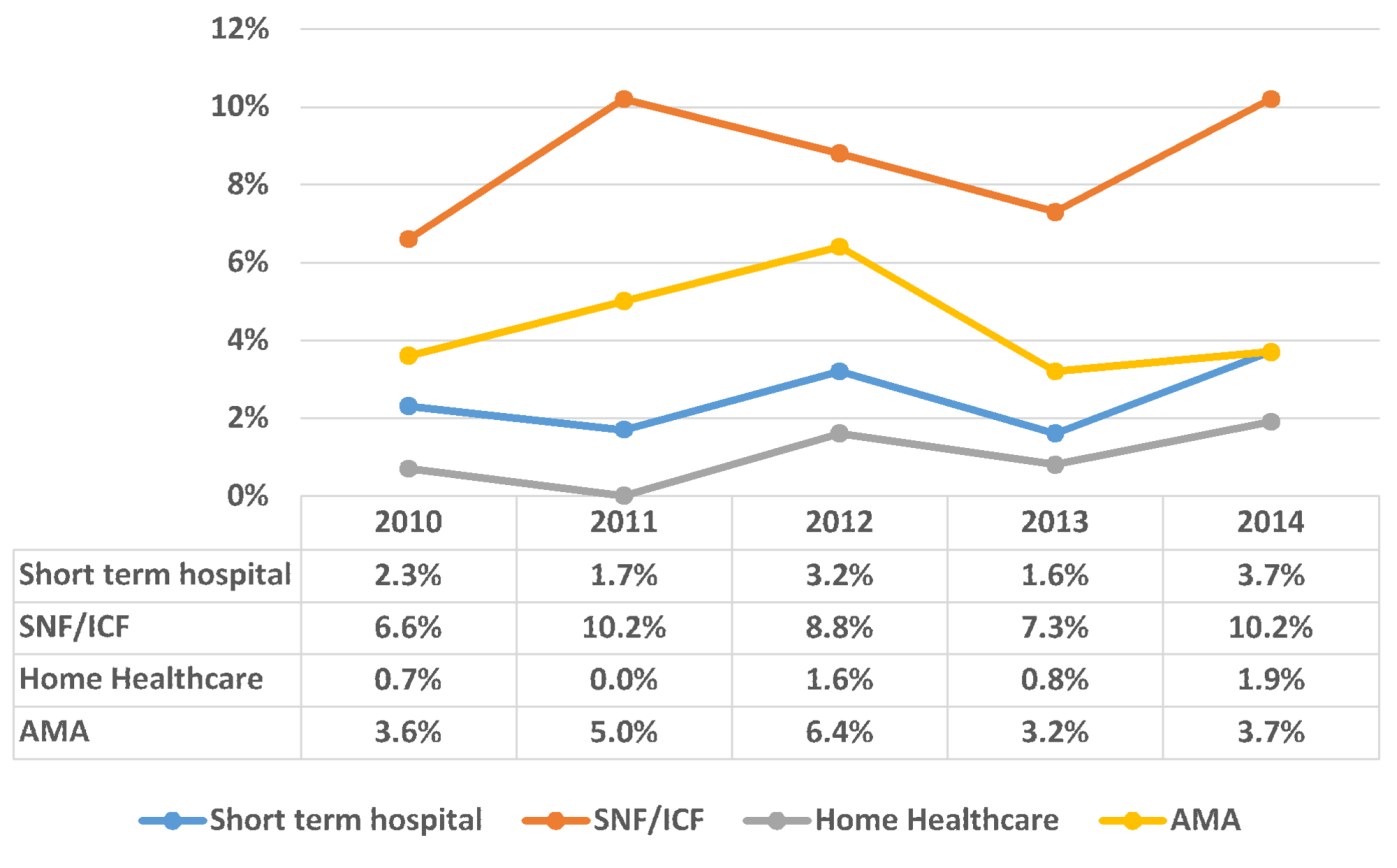
Trend of bulimia nervosa distributed according to the discharge of bulimia nervosa patients X-axis: years; Y-axis: proportion in percentage (%); Significant p-values ≤ 0.05 at 95% confidence interval. SNF: skilled nursing facility; INF: intermediate nursing facility; AMA: against medical advice

Among adverse discharge outcomes of bulimia nervosa patients, there was a variable trend in the disposition to a short-term hospital (2.4%; N = 80) and a skilled nursing facility (SNF)/intermediate care facility (ICF) (8.6 %; N = 286) (p < 0.001). The trend of bulimia nervosa from 2010–2014 was distributed according to discharge, as shown in Figure 6. 

Comorbidities in bulimia nervosa

The most prevalent psychiatric comorbidities present in bulimia nervosa were psychosis (N = 1740; 52.4%), followed by depression (N = 779; 23.5%). Drug abuse (N = 715; 21.5%) and alcohol abuse (N = 566; 17.1%) were less prevalent psychiatric comorbidities in the admitted bulimia nervosa patients.

**Figure 7:**
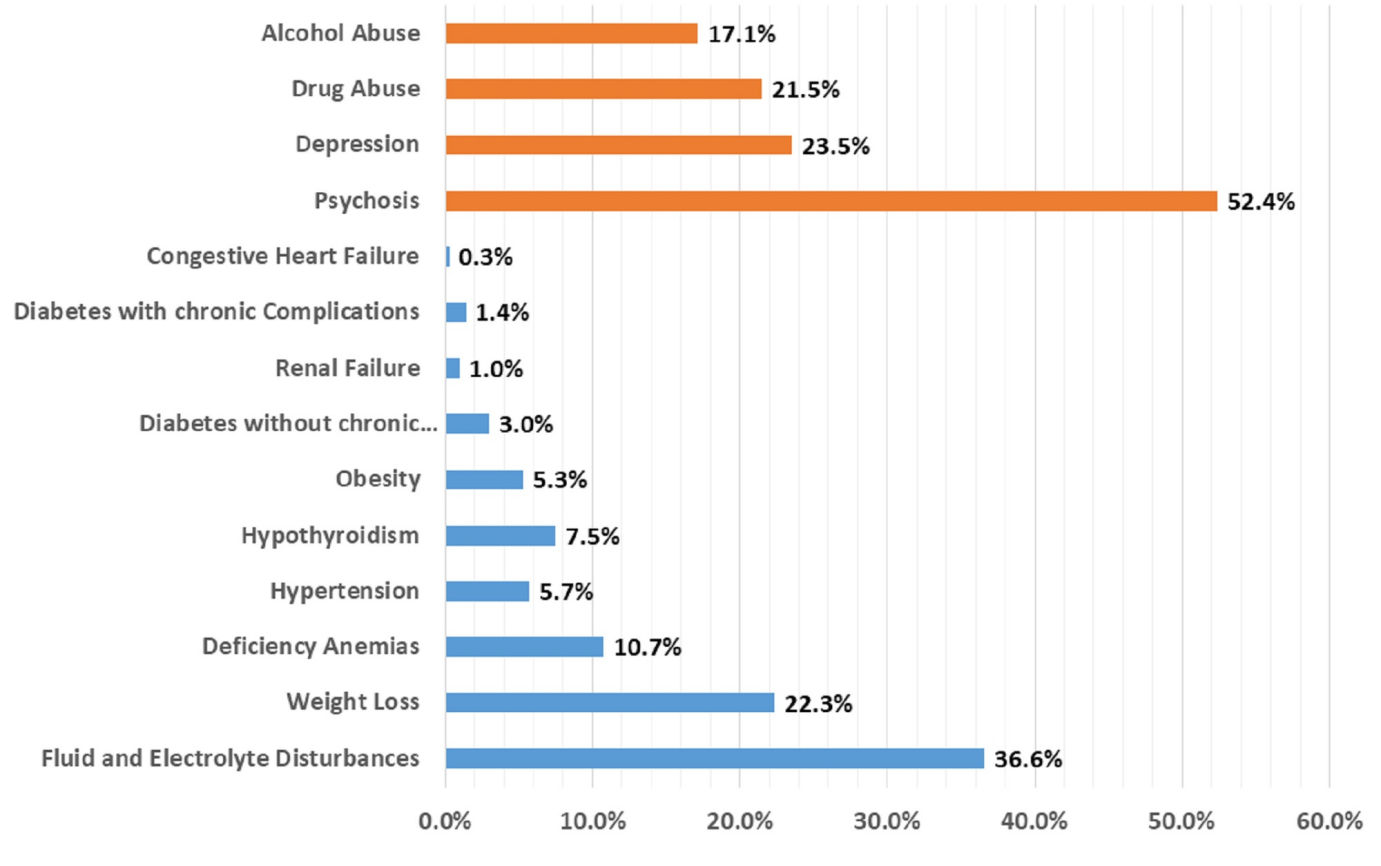
Distribution of comorbidities in bulimia nervosa patients X-axis: proportion in percentage (%); Y-axis: comorbidities. Significant p-values ≤ 0.05 at 95% confidence interval

The most prevalent medical comorbidity present in bulimia nervosa was fluid and electrolyte disorders (N = 1,214; 36.6%). The other medical co-morbidities reported among admitted patients were weight loss (N = 740; 22.3%), deficiency anemias (N = 354; 10.4%), hypothyroidism (N = 248; 7.5%), obesity (N = 177; 5.3%), hypertension (N = 190; 5.7%), uncomplicated diabetes (N = 99; 3%), diabetes with chronic complications (N = 48; 1.4%), renal failure (N = 34; 1%), and congestive heart failure (N = 10; 0.3%). The prevalence of psychiatric and medical comorbidities in bulimia nervosa patients is shown in Figure 7.

Among chronic condition comorbidities, female patients had three times higher odds of comorbid uncomplicated diabetes (OR = 3.374; 95% CI 1.899 - 5.994; p-value < 0.001), followed by hypertension (OR = 2.548; 95% CI 1.582 - 4.103; p-value < 0.001). Also, females had higher odds of psychiatric conditions, like comorbid depression (OR = 1.670; 95% CI 1.199 - 2.326; p-value = 0.002) and drug abuse (OR = 2.008; 95% CI 1.477 - 2.731; p-value < 0.001). Bulimia nervosa females also had a higher risk of fluid and electrolyte disorders during hospitalization (OR = 1.437; 95% CI 1.089 - 1.895; p-value = 0.010).

The median length of inpatient stay of bulimia nervosa patients was seven days. The odds of having a longer hospitalization with a stay of more than seven days was seen in patients with comorbid fluid and electrolyte disorders (OR = 1.816; 95% CI 1.557 - 2.118; p-value < 0.001), comorbid depression (OR = 1.745; 95% CI 1.436 - 2.120; p-value < 0.001), and comorbid drug abuse (OR = 1.192; 95% CI 0.996 - 1.426; p-value = 0.044). The odds of a longer hospitalization stay were also seen in patients with comorbid congestive heart failure but the results were not statistically significant (OR = 2.079; 95% CI 0.564 - 7.658; p-value = 0.272).

## Discussion

In our retrospective inpatient sample cohort study, we demonstrated that most of the admitted bulimia nervosa patients were White women who were under 40 years of age. The mean length of hospital stay was nine days, which is lower than the reported average inpatient stay in affective and psychotic disorders [12-14]. More than 70% of the admitted patients had a moderate morbidity. The odds of having a longer hospitalization stay was seen in bulimia nervosa patients with comorbid fluid and electrolyte disorders, comorbid depression, and comorbid drug abuse. There were no bulimia nervosa-related inpatient deaths between 2010–2014, which is not surprising given that the likelihood of dying was reported to be minor among 82.3% of the admitted patients. The present study demonstrated the pervasiveness of psychiatric and medical comorbidities in patients admitted with a diagnosis of bulimia nervosa. The most prevalent psychiatric and medical comorbidities were psychosis and fluid and electrolyte disturbances, respectively.

A nationally representative survey conducted in the United States reported that 95% of the patients with bulimia nervosa had at least one comorbid disorder and 64% had three or more comorbid disorders [3]. In our study, psychosis, depression, and drug abuse emerged as the most common psychiatric comorbidities of bulimia nervosa in admitted patients. Unipolar major depression is commonly observed in patients with bulimia nervosa and usually begins after the onset of bulimia nervosa [3, 15]. The lifetime rate for unipolar depression in patients with bulimia nervosa (79%) exceeds the rate in the general population (23%) [15-16]. Previous research has shown that emotional dysregulation often occurs in bulimia nervosa, which might contribute to the bulimia nervosa patient's propensity for depression [17]. Patients with bulimia nervosa are prone to impulsive or compulsive injury (skin cutting or burning with a lit cigarette) [18]. A study found that all-cause mortality in bulimia nervosa was roughly three times greater than all-cause mortality for bipolar disorder and depression. However, as per our study, there was no reported inpatient death among patients with bulimia nervosa between 2010–2014. The comorbidity of bulimia nervosa with psychosis is less investigated than their comorbidity with mood and anxiety disorders. A previous study that examined psychosis in patients with bulimia nervosa did not report any case of comorbid schizophrenia, although symptoms of psychosis were reported [19]. Major depression is the most common comorbidity, followed by anxiety disorders, including generalized anxiety disorder, panic disorder, obsessive-compulsive disorder, social phobia, and posttraumatic stress disorder in nearly 60% of bulimia nervosa patients. The onset of bulimia classically occurs in adolescence or young adulthood [20]. Comorbid psychosis in young adults and adolescents related to such anxiety disorders is common, so it is very rare to have psychosis because of schizophrenia that was observed with bulimia nervosa patients in our study. The lifetime prevalence of an alcohol use disorder in patients with bulimia nervosa was estimated to be 46% [21]. A previous study that investigated the neurobiological and clinical variables associated with alcohol abuse in bulimia nervosa suggested that hypercortisolism and depressive symptoms seen in patients with bulimia nervosa could explain the higher occurrence of alcohol use disorder in this patient population [22]. Illicit drug use in patients with bulimia nervosa was reported to be 26% in a national comorbidity survey [3]. A previous study that examined the rates of substance abuse in bulimia nervosa reported that the rate of substance use was elevated among women with a history of sexual or physical abuse as compared to women without such a history. The authors suggested that the higher occurrence of substance use in bulimia nervosa may not be related uniquely to the bulimia nervosa diagnostic status but may be related to characteristics shared by women with bulimia nervosa, such as a history of sexual or physical abuse [23]. In our study, we reported females with bulimia nervosa had higher odds of psychiatric conditions, like comorbid depression and drug abuse.

Medical complications of bulimia nervosa depend upon the method and frequency of purging [24]. Self-induced vomiting was reported to cause most medical complications. As per our study, the most commonly reported medical comorbidities were electrolyte disturbances, weight loss, and deficiency anemias. The most common medical complications of bulimia nervosa related to electrolyte disturbances include dehydration, hypokalemia, hyponatremia, and metabolic alkalosis [25]. Hypokalemia in otherwise healthy young adults is highly specific for bulimia nervosa and hypokalemia-related cardiac arrhythmias can explain the increased mortality rate observed in patients with bulimia nervosa [26]. Observed weight loss in patients with bulimia nervosa might result from the high frequency of vomiting seen in these patients [27]. In our study, among medical comorbidities, female patients had higher odds of comorbid uncomplicated diabetes, hypertension, and electrolyte disturbances.

This dataset is subject to minimal reporting bias, and all information is coded independently of the individual practitioner, making it a potentially more reliable source. The limitation for using hospitalization (and not the patient) as the unit of analysis is that it does not translate to generalizability for all patients with bulimia nervosa. There may have been underreporting of chronic comorbidities in the NIS data because the administrative database was used. Hence, clinical data were not incorporated in the data source. We recommend that future research examine the influence of psychiatric comorbidities with clinical data. In addition to that, we recommend that future research should investigate the impact of different medical and psychiatric comorbidities of bulimia nervosa on admission outcomes.

## Conclusions

Our study established the psycho-socio-demographic characteristics and hospitalization outcomes of bulimia nervosa patients, including disease severity, mortality, length of inpatient stay, and total inpatient charges. In addition to that, we demonstrated the rates of the most common medical and psychiatric comorbidities of bulimia nervosa. Patients with bulimia nervosa can present with medical complications and attempt to hide their eating disorder from the clinicians. We believe that medical and psychiatric comorbidities of bulimia nervosa should be carefully investigated by clinicians as they can further complicate the management of bulimia nervosa and result in adverse inpatient outcomes.
